# Short‐Term Effects of Carbon Monoxide on Morbidity of Chronic Obstructive Pulmonary Disease With Comorbidities in Beijing

**DOI:** 10.1029/2022GH000734

**Published:** 2023-03-27

**Authors:** Zhiwei Li, Feng Lu, Mengmeng Liu, Moning Guo, Lixin Tao, Tianqi Wang, Mengyang Liu, Xiuhua Guo, Xiangtong Liu

**Affiliations:** ^1^ School of Public Health Capital Medical University Beijing China; ^2^ Beijing Municipal Key Laboratory of Clinical Epidemiology Beijing China; ^3^ Beijing Municipal Health Commission Information Centre Beijing China; ^4^ National Institute for Data Science in Health and Medicine Capital Medical University Beijing China; ^5^ School of Public Health Hebei Medical University Shijiazhuang China; ^6^ Centre for Precision Health School of Medical and Health Sciences Edith Cowan University WA Joondalup Australia

**Keywords:** CO, COPD, comorbidity, admission

## Abstract

The association between CO and chronic obstructive pulmonary disease (COPD) has been widely reported; however, the association among patients with type 2 diabetes mellitus (T2DM) or hypertension has remained largely unknown in China. Over‐dispersed generalized additive model was adopted to quantity the associations between CO and COPD with T2DM or hypertension. Based on principal diagnosis, COPD cases were identified according to the International Classification of Diseases (J44), and a history of T2DM and hypertension was coded as E12 and I10‐15, O10‐15, P29, respectively. A total of 459,258 COPD cases were recorded from 2014 to 2019. Each interquartile range uptick in CO at lag 03 corresponded to 0.21% (95%CI: 0.08%–0.34%), 0.39% (95%CI: 0.13%–0.65%), 0.29% (95%CI: 0.13%–0.45%) and 0.27% (95%CI: 0.12%–0.43%) increment in admissions for COPD, COPD with T2DM, COPD with hypertension and COPD with both T2DM and hypertension, respectively. The effects of CO on COPD with T2DM (*Z* = 0.77, *P* = 0.444), COPD with hypertension (*Z* = 0.19, *P* = 0.234) and COPD with T2DM and hypertension (*Z* = 0.61, *P* = 0.543) were insignificantly higher than that on COPD. Stratification analysis showed that females were more vulnerable than males except for T2DM group (COPD: *Z* = 3.49, *P* < 0.001; COPD with T2DM: *Z* = 0.176, *P* = 0.079; COPD with hypertension: *Z* = 2.48, *P* = 0.013; COPD with both T2DM and hypertension: *Z* = 2.44, *P* = 0.014); No statistically significant difference could be found between age groups (COPD: *Z* = 1.63, *P* = 0.104; COPD with T2DM: *Z* = 0.23, *P* = 0.821; COPD with hypertension: *Z* = 0.53, *P* = 0.595; COPD with both T2DM and hypertension: *Z* = 0.71, *P* = 0.476); Higher effects appeared in cold seasons than warm seasons on COPD (*Z* = 0.320, *P* < 0.001). This study demonstrated an increased risk of COPD with comorbidities related to CO exposure in Beijing. We further provided important information on lag patterns, susceptible subgroups, and sensitive seasons, as well as the characteristics of the exposure‐response curves.

## Key Points

- Exposure to CO was associated with an increased risk of chronic obstructive pulmonary disease (COPD) with comorbidities
- Females were more vulnerable to CO exposure among COPD with comorbidities
- Higher effect appeared in cold season than warm season among COPD group

## 1 Introduction

Acute exacerbation of chronic obstructive pulmonary disease (COPD), defined as “an acute worsening of respiratory symptoms that result in additional therapy,” is the main cause of high hospitalization rates and mortality among patients with respiratory diseases (Whittaker Brown & Braman, 2020). COPD is usually linked with several chronic diseases, such as type 2 diabetes mellitus (T2DM), cardiovascular disease, especially hypertension, which are the most common comorbidities (Wielscher et al., 2021). T2DM is present in 22%–40% of patients hospitalized with COPD (Rambaran et al., 2019). The prevalence of T2DM is higher in patients with acute exacerbation of chronic obstructive pulmonary disease (AECOPD) than in the general population. In addition, COPD patients with T2DM are associated with hypertension (Lin et al., 2021). Comorbidities can lead to disease progression and therefore play an important role in the prognosis of COPD (Vogelmeier et al., 2017).

In recent years, air pollution, as an emerging risk factor for morbidity and mortality of COPD, has attracted much attention (Renzi et al., 2022). The adverse effects of particular matters on COPD (Lee et al., 2021; Pothirat et al., 2021), T2DM (Song et al., 2018) and hypertension (Qin et al., 2021) patients have been widely reported. Carbon monoxide (CO) is a minor component of air pollution, but as a pollutant that is not easily perceived by the human senses, it can have significant health effects. High concentration of CO exposure will affect the function of heart and nervous system and bring adverse effects on human health, which should be paid enough attention. Short‐term exposure to CO has been positively associated with COPD‐related emergency department visits, hospital admissions, and mortality (Boehm et al., 2021; Chen et al., 2020; Du et al., 2021; Qu et al., 2019; Wang et al., 2021). However, the effect of CO on COPD comorbidities has not been comprehensively reported. In this study, we examined the association between short‐term exposure to CO and morbidity of COPD with comorbid T2DM or hypertension in Beijing from 2014 to 2019. In addition, we further explored whether COPD patients with diabetes and/or hypertension were more vulnerable to adverse effects due to CO exposure.

## 2 Material and Methods

### 2.1 Data Collection

Beijing, the capital city of the People's Republic of China, is situated at the northern tip of the North China Plain, at 39°56′N and 116°20′E region. The total area of the city is about 16,410.54 square kilometers, including 16 districts, with a population of 21.89 million by the end of 2020.

Admission data were obtained from the Information Center of Beijing Municipal Health Commission (http://www.phic.org.cn/). The data mainly included the time of admission, gender, age, main diagnosis and secondary diagnosis of COPD patients admitted to 258 public hospitals in 16 districts in Beijing from 1 January 2014 to 31 December 2019. The study population was divided into four groups based on whether hospitalized patients with COPD were simultaneously diagnosed with T2DM and/or hypertension. According to the 10th revision of the International Classification of Diseases (ICD‐10), COPD was coded as J44.0‐J44.1. T2DM was coded as E12, which was defined as fasting blood glucose ≥7.1 mmol/L, and/or current treatment of diabetes with antidiabetic medication before admission. Hypertension (ICD‐10: I10‐15, O10‐15, P29) was defined as systolic blood pressure ≥140 mmHg and/or diastolic blood pressure ≥90 mmHg and/or taking antihypertensive agents. Daily hospital admissions were further categorized by gender, age (≤60; >60 years of age). This study only included hospitalized cases of COPD in Beijing residents. The data recording system in the study area has been proven to be of high validity (Aklilu et al., 2020; M. Liu et al., 2021; X. Liu et al., 2021).

Hourly concentrations of ambient CO and fine particulate matter (PM_2.5_), inhalable particulate matter (PM_10_), nitrogen dioxide (NO_2_), sulfur dioxide (SO_2_), ozone (O_3_) from 35 stations in 16 districts in Beijing were retrieved from the Beijing Environmental Protection Bureau during the same period. In this study, we averaged air pollution data as daily levels. Given that meteorological parameters may alter the associations between air pollution exposure and COPD, hourly data of temperature (°C) and relative humidity (%) of 18 meteorological monitoring stations in Beijing were obtained from online China Meteorological Data Sharing Service System online over the study period.

### 2.2 Ethical Clearance

The study was approved by the Institutional Review Board of Capital Medical University (No. IRB00009511). Informed consent was not speciﬁcally required since personal identiﬁers were not collected.

### 2.3 Statistical Analysis

As the daily admissions generally followed a Poisson distribution, the effects of CO on COPD hospital admissions were investigated using an over‐dispersed generalized additive model, Model 1 as follows:

$$\log\left[E\left(Yt\right)\right]=intercept+\beta Z_{t}+s\left(\;time,7/year\right)+s\left(temp,3\right)+s\left(RH,3\right)+{DOW}_{t}+{Holiday}_{t}$$

where E(Yt) represents the number of hospital admissions for COPD on dayt; Zt is the CO concentration on day t; β represents the log‐relative risk (RR) of COPD admissions associated with an interquartile range (IQR) increase in CO concentration; s() indicates a natural spline function to filter out long‐term trends and seasonal patterns in daily COPD admissions; temp is the daily mean temperature (℃); RH is the relative humidity $\left(\%\right);{DOW}_{t}$ is the day of the week; and ${DOW}_{t}$ and ${Holiday}_{t}$ were included as categorical variables. Considering that a nonlinear relationship has been shown between temperature, relative humidity and hospitalizations for COPD in the previous studies (Tian, Xiang et al., 2018; Wang et al., 2021), a natural cubic spline with 3 degrees of freedom (*df*) was used for each weather condition variable.

We created a binary variable for season, with 0 for warm season (from May to October) and 1 for cold season (November to April). Then we added a product term between pollutant concentrations and season into the core model to test the possible interaction between air pollution and season (Guo et al., 2010). Model 2 as follows:

$$\log\left[E\left(Yt\right)\right]=intercept+\beta_{1}Z_{t}+\beta_{2}season+\beta_{3}Z_{t}+s\left(\;time,7\right)+s\left(temp,3\right)+s\left(RH,3\right)+{DOW}_{t}+{Holiday}_{t}$$

$\beta_{1}$ signifies the main effect of the pollutant in the warm season; ($\beta_{1}+\beta_{3}$) was the pollutant effect in the cold season; $\beta_{2}$ is a vector for coefficients of the season, and $\beta_{3}$ is a vector for coefficient of the interactive term between pollutant and season.

Sensitivity analysis was conducted to check the stability of the model. First, we checked the *df* values of the time variable from 4 to 10 per year. Moreover, we also conducted bi‐pollutant model by including PM_2.5_, PM_10_, NO_2_, SO_2_, and O_3_ one at a time into the model and changed the number of *df* values of the time variable from 4 to 10 per year to assess the robustness of the effect estimate.

Given the uncertainty in determining the best lag days for estimation, we used multiple lag structures, including single‐day lags from 0 to 3 and moving average exposures from multiple days. A Z value was calculated to test the statistical significance of subgroup differences as follows: $Z=\left(\beta_{1}-\beta_{2}\right)/\sqrt{{SE}_{1}^{2}+{SE}_{2}^{2}}$, where $\beta_{1}$ and $\beta_{2}$ were the effect estimates for the two categories (e.g., males and females) and ${SE}_{1}$ and ${SE}_{2}$ were the corresponding standard errors (Altman & Bland, 2003). Then, a *P* value could be obtained from the standard normal distribution based on the Z value. Spearman's correlation coefficients were calculated to assess the degrees of correlation between air pollutants and meteorological variables. The statistical tests were two‐sided, and associations with *P* < 0.05 were considered statistically significant. The effects are described as the percentage change and 95% confidence interval (CI) in daily counts of admissions for COPD per IQR increase in air pollutants. All statistical models were run in R software (version 4.0.2) using the *mgcv* package.

## 3 Results

### 3.1 Descriptive Analyses

Table 1 summarized the descriptive statistics of daily admissions in COPD, stratified by gender, age and season. In total, 459,258 admissions for COPD were included in this study during the period from 2014 to 2019, of which 121,330 patients with comorbid both T2DM and hypertension (accounting for 26.42%). The majority of patients were male (67.34%) and over 60 years old (93.09%). The annual average values of daily mean concentrations of air pollutants were 0.99 mg/m^3^ for CO, 65.94 μg/m^3^ for PM_2.5_, 98.52 μg/m^3^ for PM_10_, 42.91 μg/m^3^ for NO_2_, 9.24 μg/m^3^ for SO_2_ and 60.77 μg/m^3^ for O_3_. The annual average values were 12.41°C for temperature and 53.52% for relative humidity (Table S1 in Supporting Information S1).

**Table 1:** Characteristics and Distribution of Chronic Obstructive Pulmonary Disease Events

	COPD		COPD with hypertension		COPD with T2DM		COPD with both T2DM and hypertension		Total	
	No.	(%)	No.	(%)	No.	(%)	No.	(%)	No.	(%)
Gender										
Male	128,546	69.66	27,320	63.77	72,916	65.96	80,491	66.34	309,273	67.34
Female	55,987	30.34	15,524	36.23	37,635	34.04	40,839	33.66	149,985	32.66
Age (years)										
≤60	15,436	8.36	2826	6.60	6,154	5.57	7,302	6.02	31,718	6.906
>60	169,097	91.64	40,018	93.40	104,397	94.43	114,028	93.98	427,540	93.09
Season										
Warm	83,596	45.30	19,378	45.23	49,487	44.76	54,463	44.89	206,924	45.06
Cold	100,937	54.70	23,466	54.77	61,064	55.24	66,867	55.11	252,334	54.94
Total	184,533	40.18	42,844	9.33	110,551	24.07	121,330	26.42	459,258	100

*Note.* COPD: acute exacerbation of chronic obstructive pulmonary disease; T2DM: type 2 diabetes mellitus; No. (%) ‐counts of event and percentage.

Figure S1 in Supporting Information S1 showed pairwise Spearman correlation coefficients between air pollutants and weather conditions. Air pollutants except O_3_ were strongly correlated with each other (Spearman's correlation coefficients distributed from 0.59 to 0.89) and were moderately correlated with weather conditions (−0.46–0.76).

### 3.2 Association Between Pollutants and COPD Admissions

**Figure 1:**
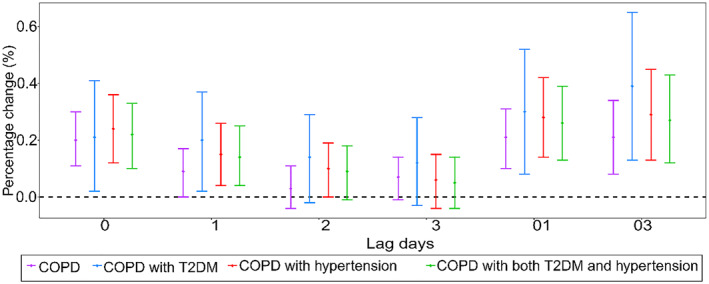
Percentage changes with 95% confidence interval in chronic obstructive pulmonary disease (COPD) admissions associated with per IQR increase in CO concentrations for different lag structures in single pollutant generalized additive model. Note: COPD: acute exacerbation of COPD; T2DM: type 2 diabetes mellitus; CO: carbon monoxide.

Figure 1 summarized the effects of CO on hospital admissions for COPD. Significant associations were found between CO and daily admissions for COPD comorbidities in Beijing. Delayed effects of CO were significantly associated with admissions on the COPD comorbidities at lag03 with the largest effect. For each IQR uptick in CO, the corresponding percentage changes were 0.21% (95%CI: 0.08%–0.34%) for COPD, 0.39% (95%CI: 0.13%–0.65%) for COPD with T2DM, 0.29% (95%CI: 0.13%–0.45%) for COPD with hypertension and 0.27% (95%CI: 0.12%–0.43%) for COPD with both T2DM and hypertension, respectively, at lag03. Specifically, the effects of CO on COPD with T2DM, COPD with hypertension, COPD with both T2DM and hypertension were higher than those in COPD group at different lag structures. However, no significant difference was found between T2DM or hypertension groups and those without comorbidities (*Z* = 1.19, *P* = 0.234; *Z* = 0.77, *P* = 0.444; *Z* = 0.61, *P* = 0.543).

### 3.3 Stratification Analyses

The associations between a IQR increase pollutants in the lag03 and the risk of COPD admissions by gender, age and season groups are presented in Table 2. CO were significantly associated with hospital admissions for COPD among female, age >60 years patients. There was significant difference between gender group in the effects of CO on the COPD comorbidities in addition to T2D group (COPD：*Z* = 3.49, *P* < 0.001; COPD with T2DM: *Z* = 0.176, *P* = 0.079; COPD with hypertension: *Z* = 2.48, *P* = 0.013; COPD with both T2DM and hypertension: *Z* = 2.44, *P* = 0.014). No statistically significant difference could be found between age groups (COPD: *Z* = 1.63, *P* = 0.104; COPD with T2DM: *Z* = 0.23, *P* = 0.821; COPD with hypertension: *Z* = 0.53, *P* = 0.595; COPD with both T2DM and hypertension: *Z* = 0.71, *P* = 0.476). The effects on admissions of COPD comorbidities were statistically significant except for COPD with T2DM group in warm season. Effect of CO on COPD was significantly higher in warm season than cold season at lag03 (*Z* = 3.20, *P* = 0.001). We found that there was no significant difference between the seasonal groups in the COPD comorbidities group (COPD with T2DM: *Z* = 0.17, *P* = 0.866; COPD with hypertension: *Z* = 1.10, *P* = 0.272; COPD with both T2DM and hypertension: *Z* = 1.25, *P* = 0.211).

**Table 2:** Percentage Changes (95% Confidence Interval) for Chronic Obstructive Pulmonary Disease Associated With per IQR Uptick in Exposure to CO in Beijing Among Subgroups Stratified by Gender, Age and Season

Group	Gender			Age (years)			Season		
	Male	Female	*P*	≤60	>60	*P*	Warm	Cold	*P*
COPD	0.06(−0.09, 0.21)	**0.54**(**0.32, 0.77)**	**<0.001**	−0.14(−0.58, 0.30)	**0.24(0.11, 0.38)**	0.104	**0.73(0.38, 1.09)**	**0.15(0.11, 0.20)**	**0.001**
COPD with T2DM	0.21(−0.12, 0.54)	**0.69**(**0.27, 1.12)**	0.079	0.28(−0.71, 1.28)	**0.40(0.13, 0.67)**	0.821	0.32(−0.41, 1.06)	**0.39(0.16, 0.62)**	0.866
COPD with hypertension	0.14(−0.06, 0.34)	**0.57**(**0.30, 0.84)**	**0.013**	0.11(−0.56, 0.79)	**0.30(0.14, 0.47)**	0.595	**0.54(0.08, 1.00)**	**0.26(0.08, 0.45)**	0.272
COPD with both T2DM and hypertension	0.15(−0.02, 0.31)	**0.48**(**0.26, 0.71)**	**0.018**	0.05(−0.57, 0.68)	**0.29(0.13, 0.45)**	0.476	**0.54(0.10, 0.98)**	**0.24(0.08, 0.40)**	0.211

*Note.* Overall cumulative effects of six pollutants lasting for 0–3 days (lag03) were estimated after controlling for public holidays, DOW, cubic spline variables (calendar day for time trends and seasonality. Statistically significant effect estimates are in bold. *P* value in bold obtained from the Z‐test for the difference of the effect estimates of pollutants between genders or age groups. COPD: acute exacerbation of chronic obstructive pulmonary disease; T2DM: type 2 diabetes mellitus; CO: carbon monoxide.

### 3.4 Exposure Response Curves

**Figure 2:**
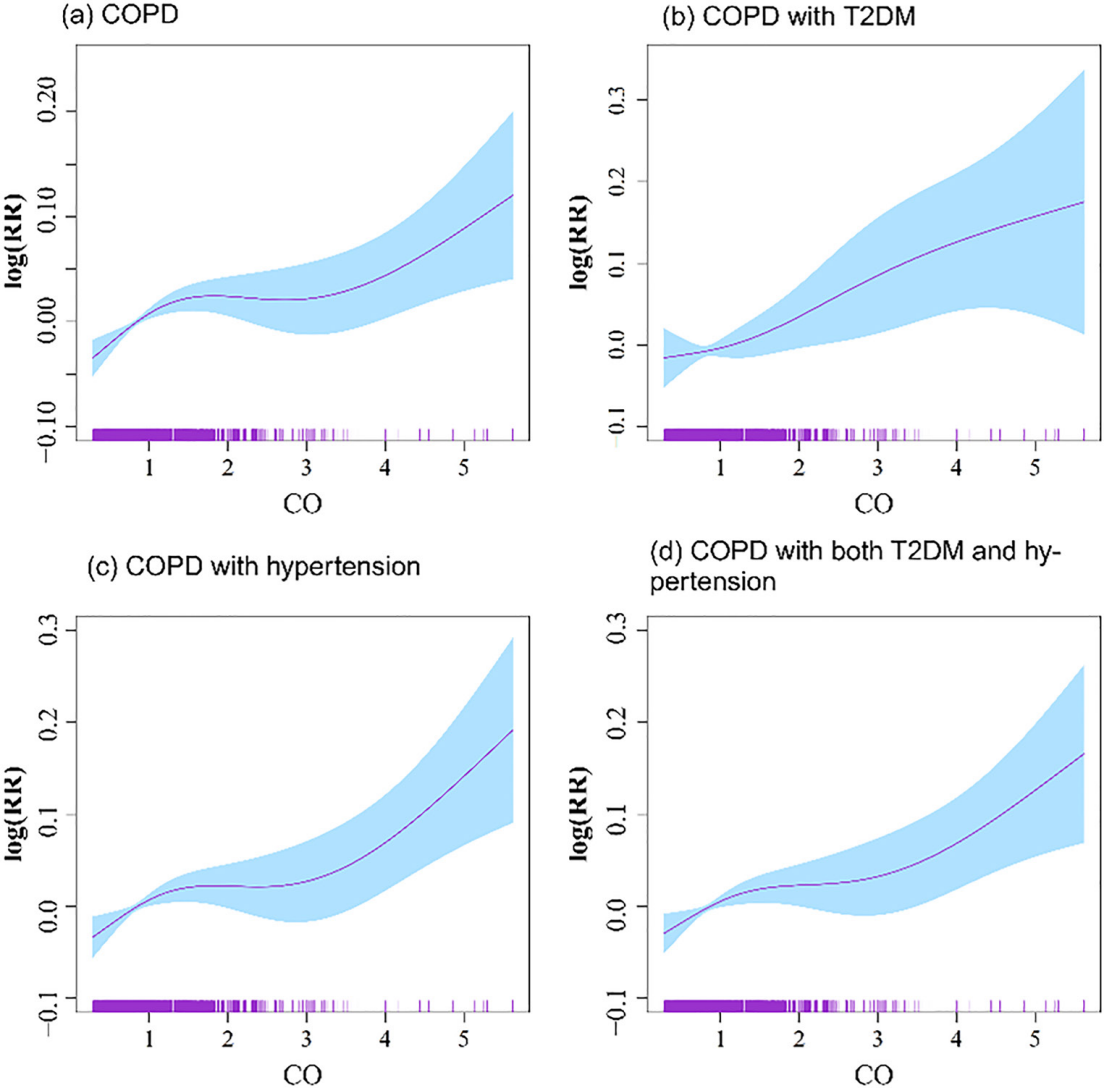
Exposure‐response curves between CO (lag03) and chronic obstructive pulmonary disease (COPD) comorbidity admissions in Beijing, 2014–2019, after adjusted for day of week and public holidays, temperature, and relative humidity. Note: COPD: acute exacerbation of COPD; T2DM: type 2 diabetes mellitus; CO: carbon monoxide.

Exposure response curves between pollutants and daily COPD comorbidity admissions were plotted in Figure 2. Four‐day moving average (lag 03) concentrations of CO appeared to have positive effect on admissions, especially for pollutants with very high concentrations. The exposure response curves of COPD, COPD with hypertension group and COPD with T2DM and hypertension were relatively flat at the concentration of 1–3 mg/m^3^ and increased sharply after exceeding 3 mg/m^3^. For COPD with T2DM group, the exposure response curves appeared to be generally linear, with an increasing trend observed.

### 3.5 Sensitivity Analysis

**Figure 3:**
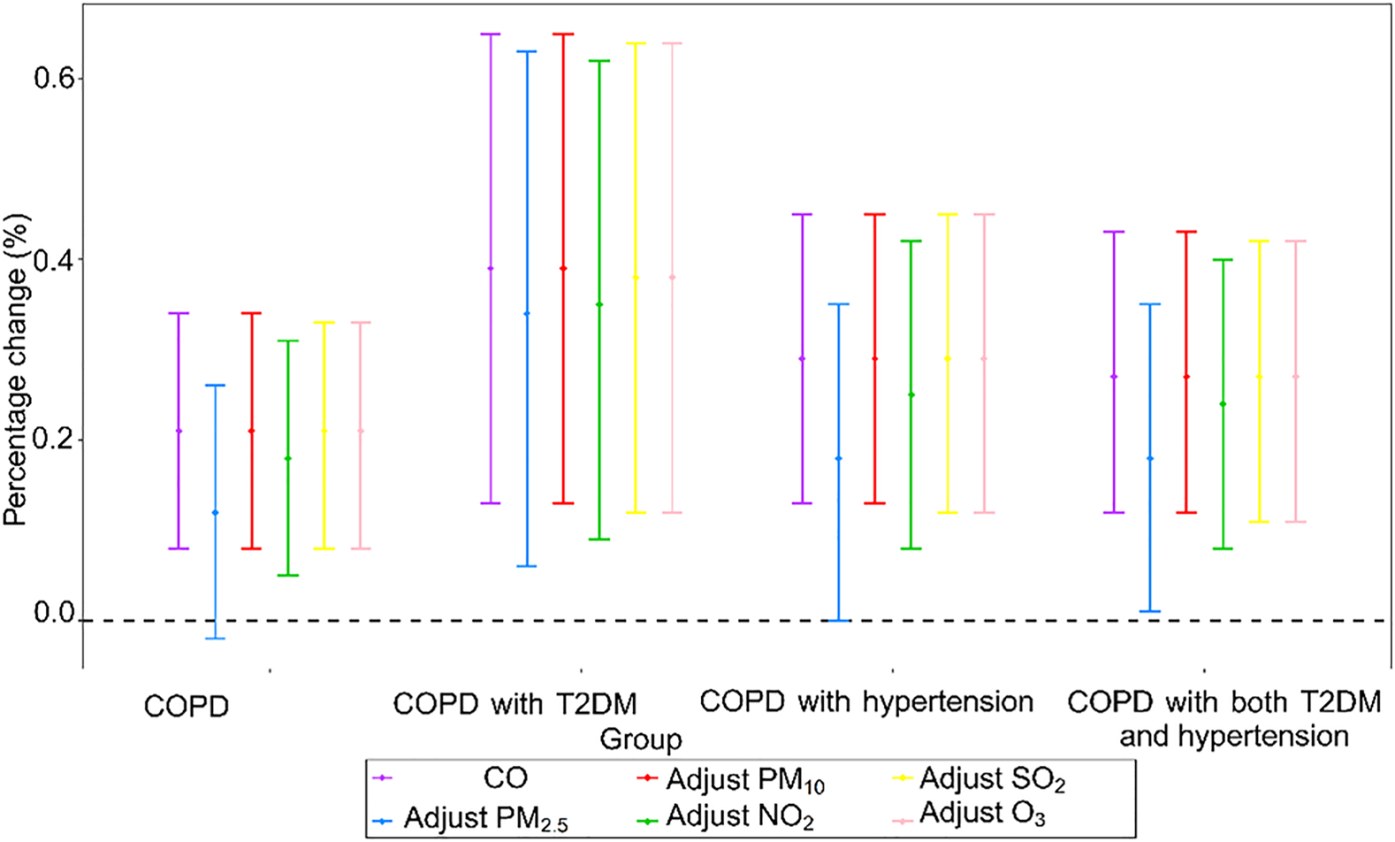
Percentage changes with 95% confidence interval in chronic obstructive pulmonary disease (COPD) admissions associated with per IQR increase in pollutants concentrations at lag03 in two‐pollutant generalized additive model. Note: COPD: acute exacerbation of COPD; T2DM: type 2 diabetes mellitus; PM_2.5_: particles with an aerodynamic diameter ≤2.5 μm; PM_10_: particles with an aerodynamic diameter ≤10 μm; NO_2_: nitrogen dioxide; SO_2_: sulfur dioxide; O_3_: carbon monoxide; CO: carbon monoxide.

**Figure 4:**
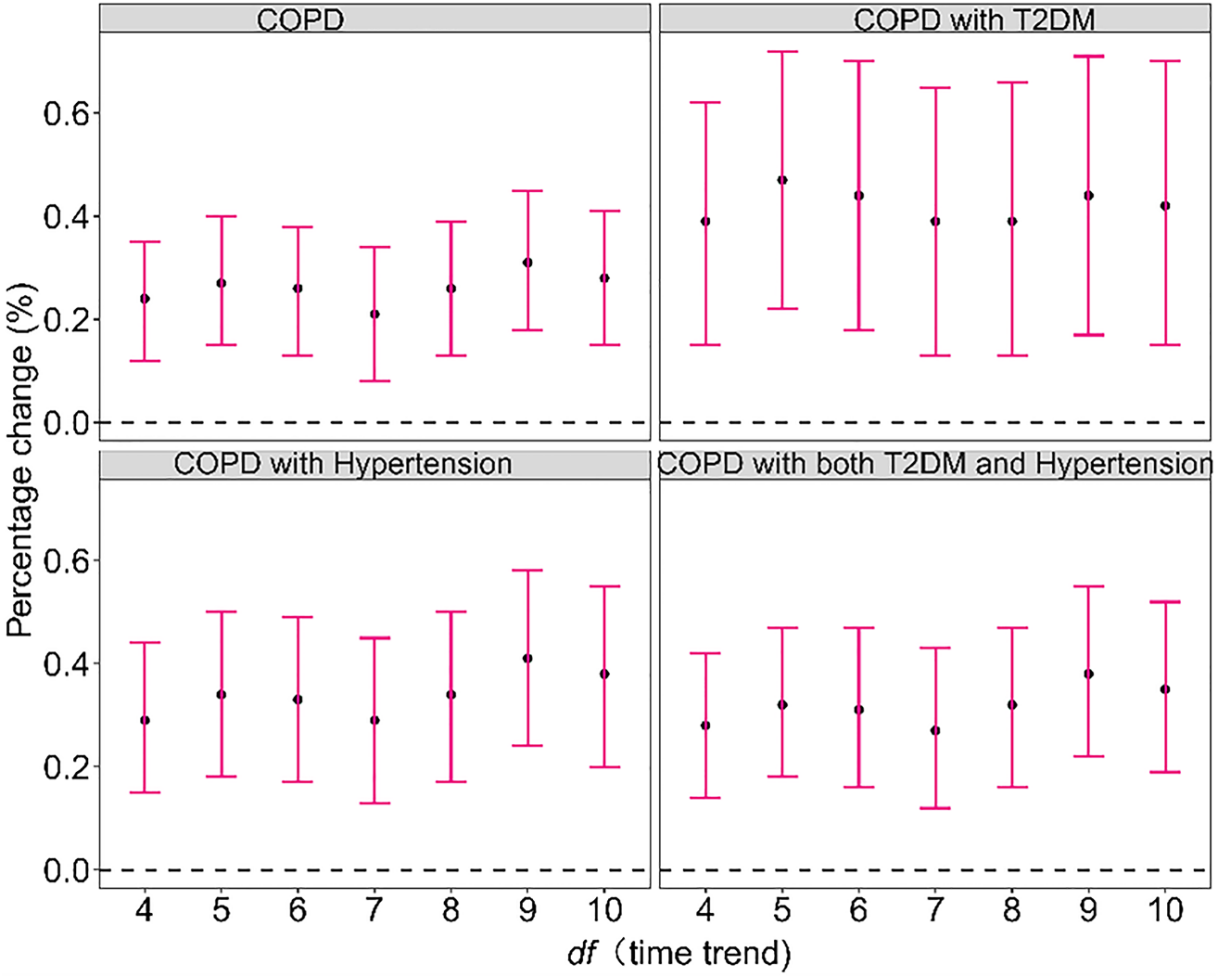
Cumulative effect (lag03) of pollutants on chronic obstructive pulmonary disease (COPD) and comorbidities for different degrees of freedom (*df*) of time trend. Note: COPD: acute exacerbation of COPD; T2DM: type 2 diabetes mellitus; CO: carbon monoxide.

After adjusting for other pollutants (Figure 3), the impact of CO on COPD admission has changed, but most of the results with statistical significance in the single pollutant model are still statistically significant in the double pollutant model. Figure 4 showed the cumulative lag effects of pollutants on the admissions of COPD complications for different *df* of time trend. The results showed that when the *df* varied in the range of 4–10, the effects of pollutants on COPD comorbidities were consistent, that is, CO were positively correlated. It can be seen that different *df* of time trend have little effect on the study of the relationship between CO and the acute effect of COPD comorbidities, and the model results of this study are relatively robust. Compared to the original model results, the results were not statistically altered when ultraviolet radiation was included as an additional covariate in the model (P values for all Z test were greater than 0.05).

## 4 Discussion

In this study, we found significant associations between short‐term exposures to CO and hospital admissions for COPD. Our results indicated that the effects of CO on COPD with T2DM, COPD with hypertension, COPD with both T2DM and hypertension were higher than those observed in COPD group at different lag days, but these differences were not statistically significant. Additionally, our findings suggest that females and individuals exposed during warm seasons may be more vulnerable to daily CO exposure.

Although prior studies have reported on the association between air pollution and COPD admissions (Boehm et al., 2021; Chen et al., 2020; Du et al., 2021; Mohebbichamkhorami et al., 2020; Sun et al., 2018, 2019), little is known about the effects of CO on the admissions of COPD comorbidities. A study conducted in Qingdao, China, on the association between air pollution and COPD showed no association between CO and COPD (Yang et al., 2021). A study conducted in Hong Kong found a possible negative association between CO and COPD (Tian et al., 2014). Another review on the health effects of gaseous pollutants, published in 2022, found only a positive association between CO and cardiovascular diseases and Parkinson's disease (Chen et al., 2022) and did not focus on the effect of CO on COPD. More evidence is needed to establish a solid association between CO and COPD comorbidities admissions. The results are consistent with previous findings that a study conducted in Beijing (Liang et al., 2019) with consistent data sources showed that in single‐pollutant models at lag0, the RR of hospitalization for COPD per IQR increase in pollutant was 1.024 (95% CI 1.018–1.029) for CO.

Our study did not find that COPD patients with T2DM and/or hypertension were more vulnerable to adverse effects from CO exposure. This may be related to the particularity of the effect of CO on human body. CO, which mainly comes from traffic pollution, is a highly toxic pollutant to the blood and nervous system. CO could enter human blood through the respiratory system and combines with hemoglobin in blood, myoglobin in muscle and respiratory enzyme containing divalent iron to form a reversible conjugate. It not only reduces the ability of blood cells to carry oxygen, but also inhibits and delays the dissociation and release of oxyhemoglobin, resulting in the necrosis of body tissues due to hypoxia. A clinical follow‐up study of 45 patients with COPD in Beijing, China showed that an increase in the quartile range of the moving average of traffic pollution exposure was associated with a significant decrease in large and small airway function at lag 7 days (Wang et al., 2022). T2DM is a common comorbidity in patients with COPD and is probably associated with increased systemic inflammation and poor outcomes. Studies have shown that COPD patients hospitalized for exacerbation are at high risk for impaired glucose metabolism (Mekov et al., 2016). COPD with diabetes was observed to be positively correlated with hypertension, suggesting that patients both with COPD and T2D were more likely to suffer from hypertension than patients with COPD (Lin et al., 2021). However, Co protects pancreas β Cells are protected from apoptosis induced by cytokines and hydrogen deficiency and promote apoptosis β Cell regeneration. CO is of great value in the treatment of T2DM and the prevention of prediabetes from developing into diabetes (Bahadoran et al., 2022).

After adjusting for other pollutants, we found that CO still had harmful effects on COPD patients with T2DM and/or hypertension. This may be related to the co‐exposure of CO with other pollutants. Although it is still unclear about the pathogenic mechanisms of co‐exposure to COPD, some pathways may explain it. A study from Italy showed that CO decreases forced expiratory volume in 1s (FEV1) and forced vital capacity (FVC) (Canova et al., 2010). Other gaseous pollutants, such as SO_2_, have also been shown to significantly reduce FEV_1_ (Ghozikali et al., 2015). Particulate pollutants inhaled by humans can be deposited in the lungs, especially in the alveoli, also decreasing FEV1 and FVC (Kyung & Jeong, 2020). In addition, particulate pollutants promote oxidative stress and induce cellular inflammation, reducing the body's immunity (Alemayehu et al., 2020). All of these can make people more susceptible to COPD.

Identification of the potentially susceptible populations is crucial to public health in developing more targeted intervention strategies. We found a larger association of air pollution in female patients, which is consistent with previous studies (Tian, Li et al., 2018). Different airways sizes, airway reactivity, lung structural and deposition of particles in the lungs between females and males may partly explain the gender differences. Another explanation is that non‐smokers may be more sensitive to air pollution than smokers, since in China, the smoking rate is much lower in females than males (Xu et al., 2016). In addition, we found similar results as previous studies that older people are more susceptible to air pollution (Guan et al., 2016; Liang et al., 2019). Due to poorer lung function and the weaker immune system, elderly patients are more likely to suffer from air pollution. These findings suggested that elderly COPD patients were most susceptible and should be intensively protected from exposure to outdoor air pollution. Furthermore, we found a stronger association during the warm season. One possible explanation is that people tend to be more active outdoors in the warm season compared to the cold season, which leads to longer exposure to CO (Zhuang et al., 2021). However, more studies are needed to confirm the seasonal patterns in the associations of air pollution on COPD and its comorbidity occurrence.

Several limitations should be noted in our study. First, we used fixed‐site monitor measurements as a proxy for personal exposure, which may result in exposure errors and an underestimation of the associations between ambient air pollution and diseases. However, measuring every participant's exposure directly is not feasible in such a large epidemiologic study. Second, the generalizability of our results might be limited, as the study collected data from only one highly‐polluted city. Third, since the date of diagnosis is not available, the accuracy of the occurrence of COPD, T2D and hypertension cannot be determined. Fourth, owing to the limited availability of data, we were unable to eliminate the influence of planned admissions.

## 5 Conclusions

Short‐term CO exposure were associated with increased admission of COPD and its comorbidity. Our study indicates that the prevention and control for COPD should be given more attention on people with T2DM or hypertension, suggesting that more efforts may be required to mitigate air pollution in Beijing, China.

**Abbreviations**
COPD: acute exacerbation of chronic obstructive pulmonary disease
T2DM: type 2 diabetes mellitus
CO: carbon monoxide
PM_2.5_: fine particulate matter with an aerodynamic diameter ≤2.5 μm
PM_10_: inhalable particulate matter with an aerodynamic diameter ≤10 μm
NO_2_: nitrogen dioxide
SO_2_: sulfur dioxide
O_3_: ozone
ICD‐10: International Classification of Diseases, 10th Revision
GAM: generalized additive model
RR: relative risk
IQR: interquartile range
*df*: degrees of freedom
E‐R curves: exposure‐response curves
95% CI: 95% confidence interval
SD: standard deviation

## Conflict of Interest

The authors declare no conflicts of interest relevant to this study.

## Supporting information

Supporting Information S1

## Data Availability

Air pollutant data and meteorological data are available at https://quotsoft.net/air/ and http://data.cma.cn, but users need to register for free on this website to access the data. Due to the Information Center of Beijing Municipal Health Commission's data policy, admission data for patients with COPD and COPD comorbidities are not available to the public. Detailed steps for downloading air pollutant and meteorological data can be found in the Supporting Information S1 “Data Download Tutorial” section.
