# Supplementary material for: Short‐Term Effects of Carbon Monoxide on Morbidity of Chronic Obstructive Pulmonary Disease With Comorbidities in Beijing
Source: Geohealth. 2023 Mar 27;7(3):e2022GH000734. doi: 10.1029/2022GH000734 (PMC10042128; doi:10.1029/2022GH000734)

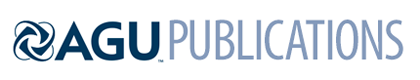


*Geo Health*

Supporting Information for

**Short-term effects of carbon monoxide on morbidity of chronic obstructive pulmonary disease with comorbidities in Beijing**

Mengmeng Liu 1, 2, 3, #, Feng Lu 4, #, Zhiwei Li 1, 2, #, Moning Guo 4, Lixin Tao1, 2, Tianqi Wang 4, Mengyang Liu 1, 2, Xiuhua Guo 1, 2, 3, 5, *, Xiangtong Liu 1, 2, *

1 School of Public Health, Capital Medical University, Beijing, 100069, China.

2 Beijing Municipal Key Laboratory of Clinical Epidemiology, Beijing, 100069, China.

3 National Institute for Data Science in Health and Medicine, Capital Medical University.

4 Beijing Municipal Health Commission Information Centre, Beijing, 100034, China.

5 Centre for Precision Health, School of Medical and Health Sciences, Edith Cowan University, Australia.

# Mengmeng Liu, Feng Lu, and Zhiwei Li contributed equally to this work.

**Contents of this file**

Figures S1

Tables S1 to S3

Data Availability Statement

**Introduction**

This supporting information includes a description of the distribution of air pollutants and meteorological factors, a correlation matrix plot, and the results of a series of generalized additive model sensitivity analyses.


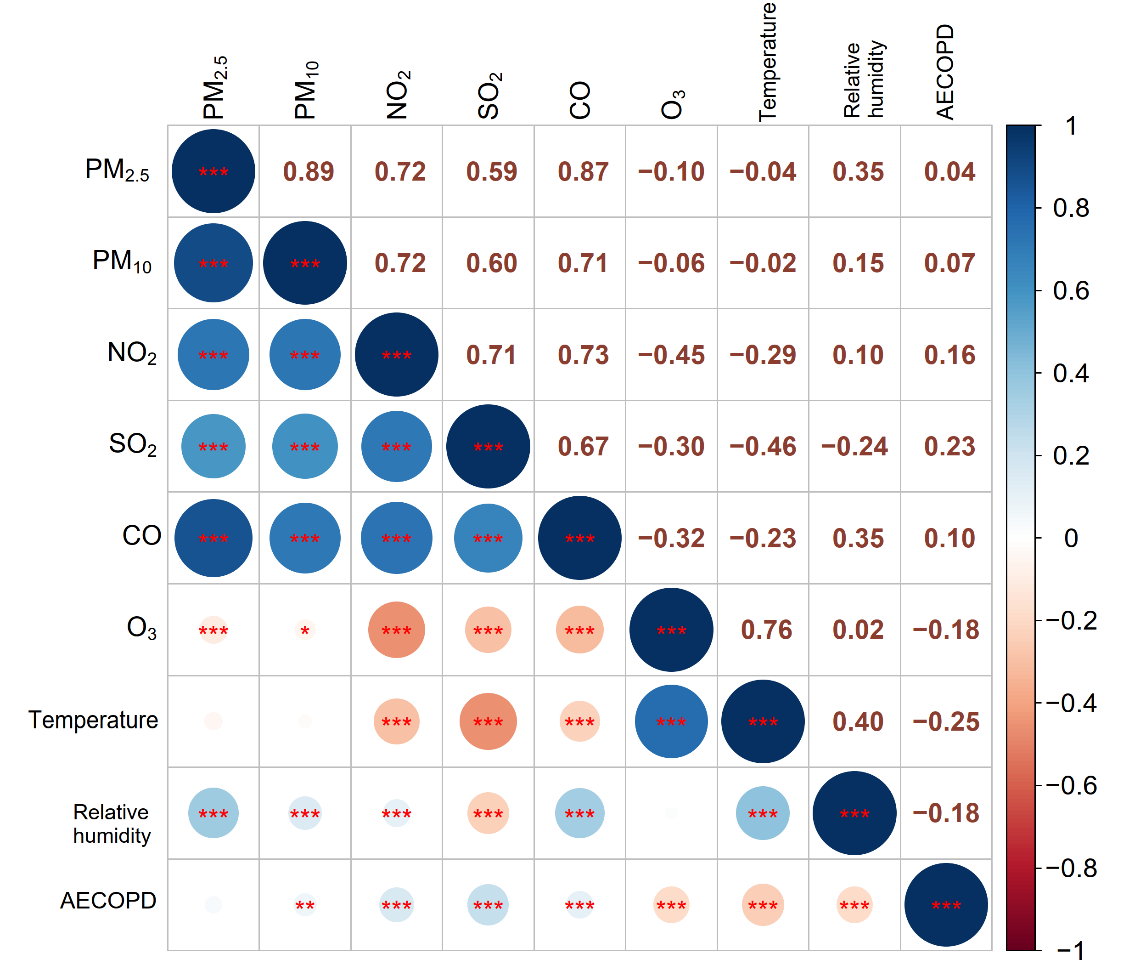


Figure S1. Correlation coefficient matrix heat map of air pollutants and weather variables.

Note: PM2.5: particles with an aerodynamic diameter ≤ 2.5 μm; PM10: particles with an aerodynamic diameter ≤ 10 μm; NO2: nitrogen dioxide; SO2: sulfur dioxide; O3: ozone; CO: carbon monoxide; COPD: acute exacerbation of chronic obstructive pulmonary disease (sum of COPD and its comorbidities).

Table S1. Summary statistics of air pollutant concentrations, weather conditions in Beijing.

| Variables | Mean±SD | Minimum | Percentile | | | Maximum | IQR |
| --- | --- | --- | --- | --- | --- | --- | --- |
|  |  |  | 25^th^ | 50^th^ | 75^th^ |  |  |
| Air pollutants |  |  |  |  |  |  |  |
| PM_2.5_ (μg/m^3^) | 65.94 ± 58.91 | 4.23 | 25.66 | 48.85 | 85.53 | 439.24 | 59.87 |
| PM_10_ (μg/m^3^) | 98.52 ± 71.11 | 5.63 | 54.28 | 81.34 | 123.53 | 909.13 | 69.25 |
| NO_2_ (μg/m^3^) | 42.91 ± 19.84 | 8.55 | 29.13 | 38.68 | 52.60 | 141.68 | 23.47 |
| SO_2_ (μg/m^3^) | 9.24 ± 9.78 | 2.02 | 3.20 | 5.76 | 10.86 | 78.06 | 7.66 |
| O_3_(μg/m^3^) | 60.77 ± 36.97 | 3.13 | 31.75 | 55.36 | 83.31 | 177.62 | 51.56 |
| CO (mg/m^3^) | 0.99 ± 0.78 | 0.20 | 0.54 | 0.80 | 1.13 | 7.64 | 0.59 |
| Meteorological variables | |  |  |  |  |  |  |
| Temperature (°C) | 12.41 ± 11.30 | -16.91 | 1.30 | 13.65 | 22.81 | 31.42 | 21.51 |
| Relative humidity (%) | 53.52 ± 19.04 | 11.21 | 38.10 | 53.31 | 69.49 | 95.30 | 31.39 |

Note: SD: standard deviation; IQR: inter-quartile range; PM2.5: particles with an aerodynamic diameter ≤ 2.5 μm; PM10: particles with an aerodynamic diameter ≤ 10 μm; NO2: nitrogen dioxide; SO2: sulfur dioxide; O3: ozone; CO: carbon monoxide

Table S2. Percentage changes with 95% CI in COPD admissions associated with per IQR increase in CO concentrations for different lag structures with or without ultraviolet radiation in the model.

| Outcome | Lag days | PC and 95% CI  (Model 1) | PC and 95% CI  (Model 2) | Z | P |
| --- | --- | --- | --- | --- | --- |
| COPD | 0 | 0.20 (0.11, 0.30) | 0.21 (0.11, 0.30) | 0.07 | 0.95 |
|  | 1 | 0.09 (0.00, 0.17) | 0.10 (0.01, 0.19) | 0.18 | 0.86 |
|  | 2 | 0.03 (-0.04, 0.11) | 0.05 (-0.03, 0.13) | 0.31 | 0.75 |
|  | 3 | 0.07 (-0.01, 0.14) | 0.09 (0.01, 0.17) | 0.43 | 0.67 |
|  | 01 | 0.21 (0.10, 0.31) | 0.22 (0.11, 0.33) | 0.14 | 0.89 |
|  | 03 | 0.21 (0.08, 0.34) | 0.25 (0.12, 0.38) | 0.41 | 0.68 |
| COPD with T2DM | 0 | 0.21 (0.02, 0.41) | 0.20 (0.01, 0.40) | -0.06 | 0.96 |
|  | 1 | 0.20 (0.02, 0.37) | 0.20 (0.03, 0.38) | 0.05 | 0.96 |
|  | 2 | 0.14 (-0.02, 0.29) | 0.15 (-0.01, 0.31) | 0.08 | 0.93 |
|  | 3 | 0.12 (-0.03, 0.28) | 0.15 (0.00, 0.31) | 0.26 | 0.79 |
|  | 01 | 0.30 (0.08, 0.52) | 0.30 (0.07, 0.53) | 0.00 | 1.00 |
|  | 03 | 0.39 (0.13, 0.65) | 0.41 (0.15, 0.68) | 0.15 | 0.88 |
| COPD with hypertension | 0 | 0.24 (0.12, 0.36) | 0.24 (0.12, 0.36) | 0.00 | 1.00 |
|  | 1 | 0.15 (0.04, 0.26) | 0.15 (0.04, 0.26) | 0.03 | 0.98 |
|  | 2 | 0.10 (0.00, 0.19) | 0.11 (0.01, 0.21) | 0.15 | 0.88 |
|  | 3 | 0.06 (-0.04, 0.15) | 0.08 (-0.02, 0.18) | 0.33 | 0.74 |
|  | 01 | 0.28 (0.14, 0.42) | 0.28 (0.14, 0.42) | 0.01 | 0.99 |
|  | 03 | 0.29 (0.13, 0.45) | 0.32 (0.15, 0.48) | 0.22 | 0.83 |
| COPD with both T2DM and hypertension | 0 | 0.22 (0.10, 0.33) | 0.22 (0.10, 0.33) | 0.01 | 0.99 |
|  | 1 | 0.14 (0.04, 0.25) | 0.15 (0.04, 0.25) | 0.03 | 0.97 |
|  | 2 | 0.09 (-0.01, 0.18) | 0.10 (0.00, 0.19) | 0.17 | 0.87 |
|  | 3 | 0.05 (-0.04, 0.14) | 0.07 (-0.02, 0.17) | 0.36 | 0.72 |
|  | 01 | 0.26 (0.13, 0.39) | 0.26 (0.13, 0.40) | 0.02 | 0.98 |
|  | 03 | 0.27 (0.12, 0.43) | 0.30 (0.14, 0.46) | 0.25 | 0.81 |

Note: PC in Model 1 are the same as in Figure 1 in manuscript, and Model 2 adds ultraviolet radiation as a covariable on the basis of Model 1. PC: Percentage change; CI: Confidence interval; COPD: acute exacerbation of chronic obstructive pulmonary disease; T2DM: type 2 diabetes mellitus; CO: carbon monoxide.

Table S3. E value for different lag structures and outcome.

| Outcome | Lag days | RR | E value | If stable |
| --- | --- | --- | --- | --- |
| COPD | 0 | 1.020 | 1.165 | Yes |
|  | 1 | 1.009 | 1.104 | Yes |
|  | 2 | 1.003 | 1.060 | Yes |
|  | 3 | 1.007 | 1.088 | Yes |
|  | 01 | 1.021 | 1.166 | Yes |
|  | 03 | 1.021 | 1.168 | Yes |
| COPD with T2DM | 0 | 1.021 | 1.170 | Yes |
|  | 1 | 1.020 | 1.162 | Yes |
|  | 2 | 1.014 | 1.132 | Yes |
|  | 3 | 1.013 | 1.125 | Yes |
|  | 01 | 1.030 | 1.208 | Yes |
|  | 03 | 1.039 | 1.241 | Yes |
| COPD with hypertension | 0 | 1.024 | 1.182 | Yes |
|  | 1 | 1.015 | 1.138 | Yes |
|  | 2 | 1.010 | 1.108 | Yes |
|  | 3 | 1.006 | 1.080 | Yes |
|  | 01 | 1.028 | 1.198 | Yes |
|  | 03 | 1.029 | 1.203 | Yes |
| COPD with both T2DM and hypertension | 0 | 1.022 | 1.172 | Yes |
|  | 1 | 1.015 | 1.136 | Yes |
|  | 2 | 1.009 | 1.102 | Yes |
|  | 3 | 1.005 | 1.076 | Yes |
|  | 01 | 1.026 | 1.191 | Yes |
|  | 03 | 1.028 | 1.196 | Yes |

Note:. The equation of RR and PC is: PC = (RR-1)*100%. E value is calculated using ggEvalue package in R software. If RR value less than E value, the result is stable. PC: Percentage change; RR: Risk ratio; COPD: acute exacerbation of chronic obstructive pulmonary disease; T2DM: type 2 diabetes mellitus; CO: carbon monoxide.

**Data Download Tutorial**

Air pollutant data and meteorological data are available at https://quotsoft.net/air/ and http://data.cma.cn, but users need to register for free on this website to access the data. Due to the Information Center of Beijing Municipal Health Commission's data policy, admission data for patients with COPD and COPD comorbidities are not available to the public.

Please follow the steps below to download air pollutant data:

Step 1


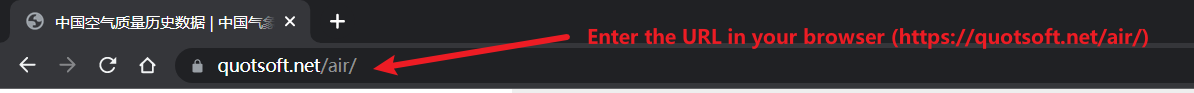


Step 2


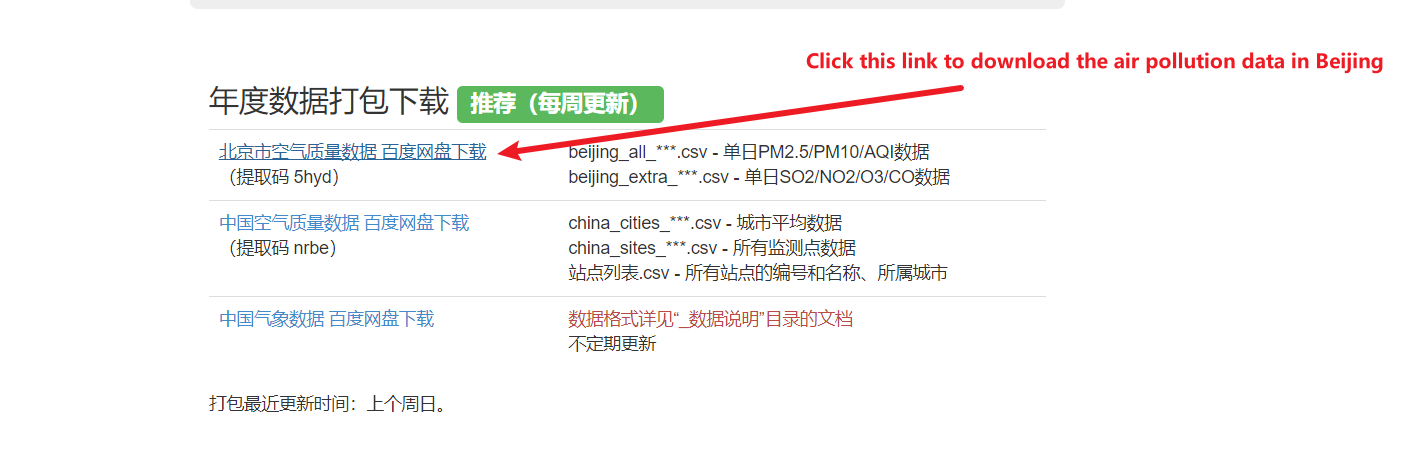


Step 3


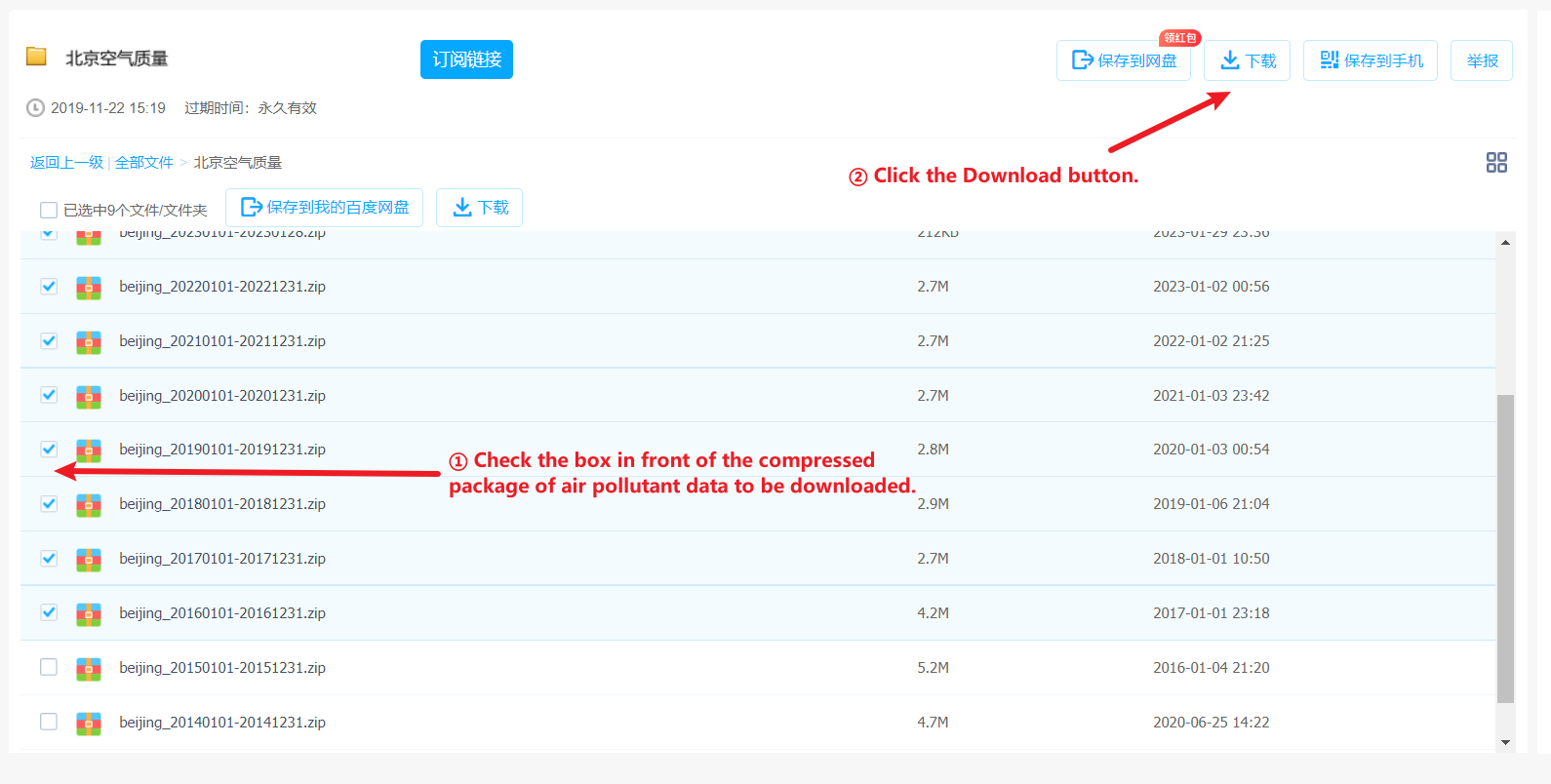


Please follow the steps below to download the meteorological data:

Step 1


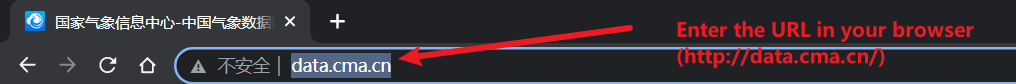


Step 2


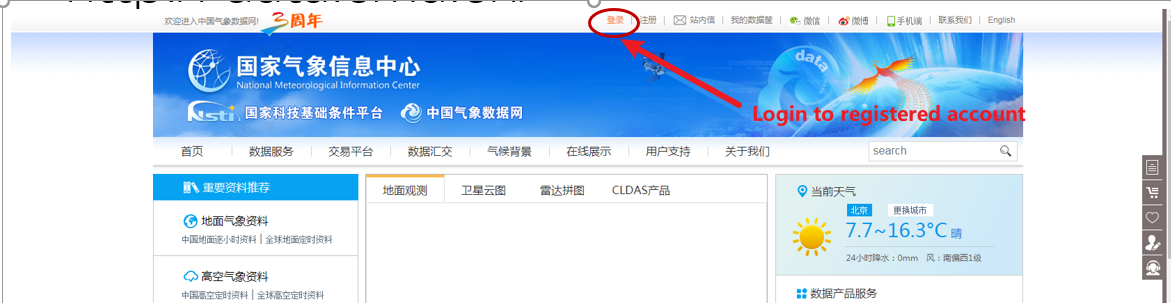


Step 3


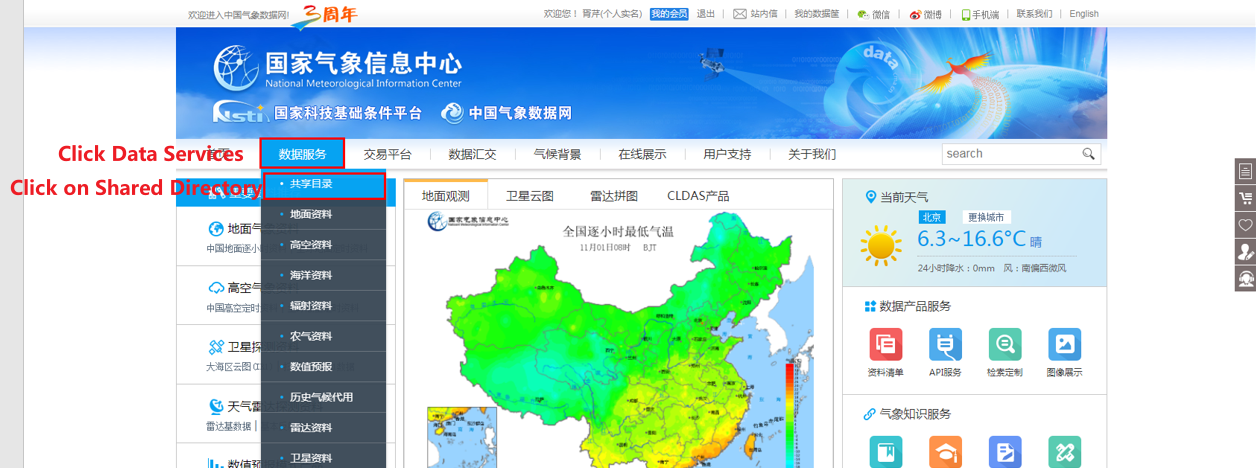


Step 4


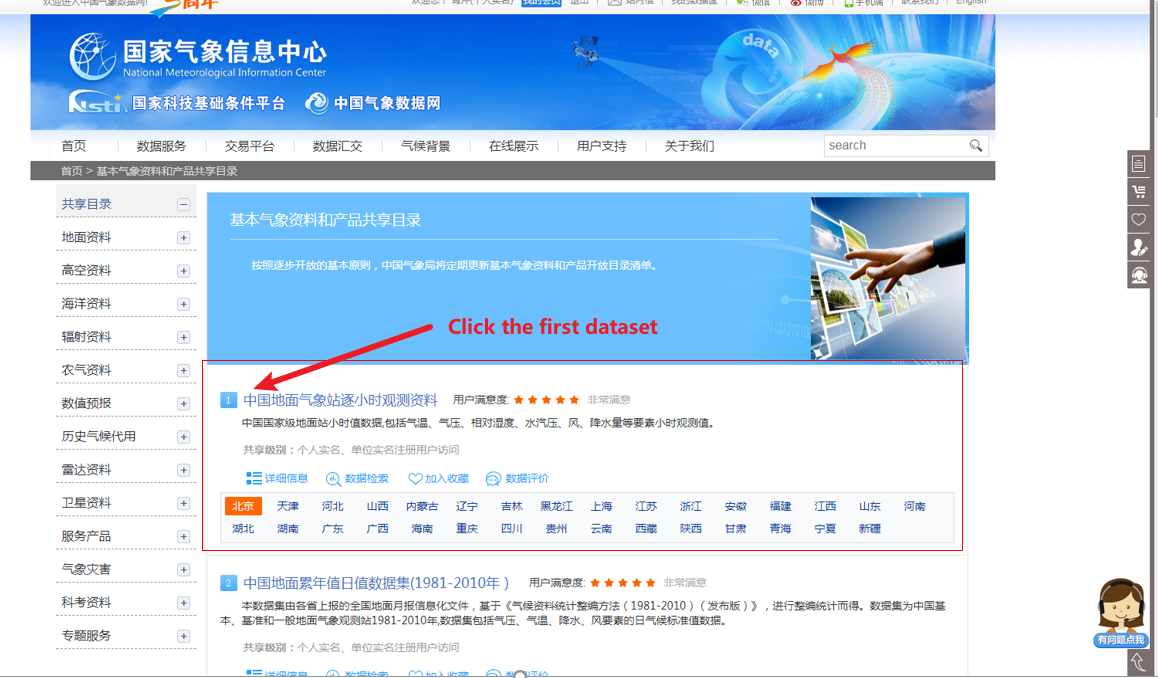


Step 5


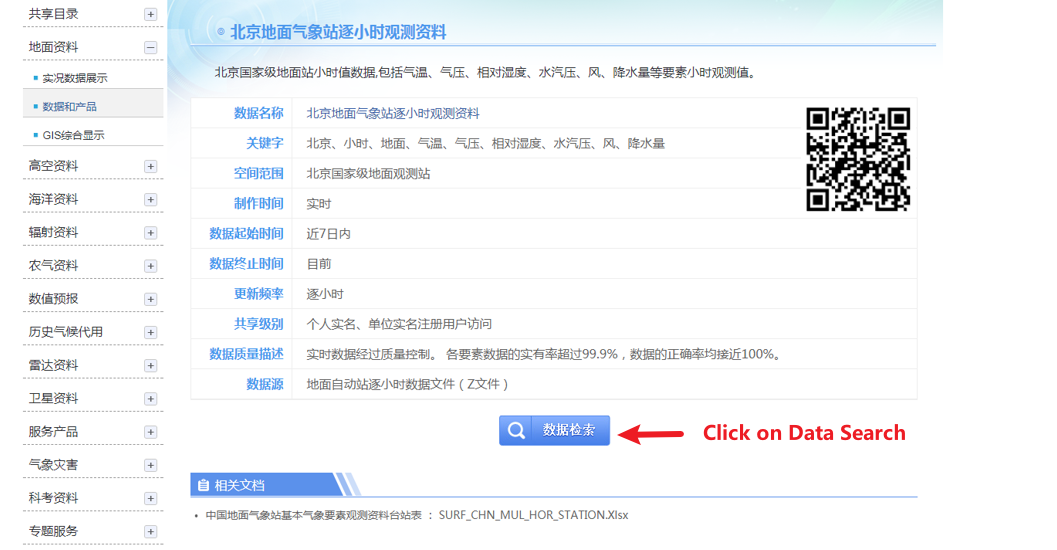


Step 6


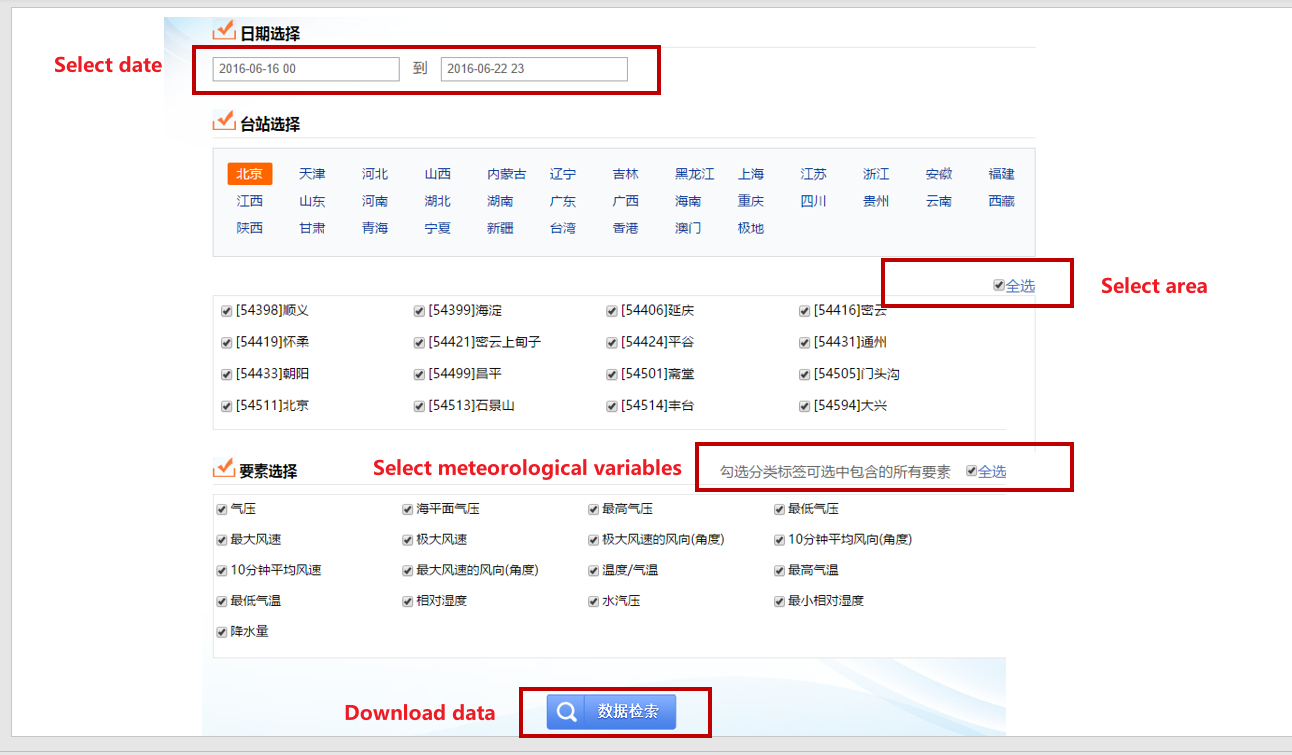

Supplement: Supplementary file 1 — Supporting Information S1 [file GH2-7-e2022GH000734-s001.docx]
